# Auditory Neuropathy as the Initial Phenotype for Patients With *ATP1A3* c.2452 G > A: Genotype–Phenotype Study and CI Management

**DOI:** 10.3389/fcell.2021.749484

**Published:** 2021-10-08

**Authors:** Wenjia Wang, Jin Li, Lan Lan, Linyi Xie, Fen Xiong, Jing Guan, Hongyang Wang, Qiuju Wang

**Affiliations:** ^1^College of Otolaryngology, Head and Neck Surgery, Institute of Otolaryngology, Chinese PLA General Hospital, Beijing, China; ^2^National Clinical Research Center for Otolaryngologic Diseases, Beijing, China

**Keywords:** auditory neuropathy (AN), *ATP1A3*, *de novo*, CAPOS syndrome, cochlear implantation (CI)

## Abstract

**Objective:** The objective of this study is to analyze the genotype–phenotype correlation of patients with auditory neuropathy (AN), which is a clinical condition featuring normal cochlear responses and abnormal neural responses, and *ATP1A3* c.2452 G > A (p.E818K), which has been generally recognized as a genetic cause of cerebellar ataxia, areflexia, pes cavus, optic atrophy, and sensorineural hearing loss (CAPOS) syndrome.

**Methods:** Four patients diagnosed as AN by clinical evaluation and otoacoustic emission and auditory brainstem responses were recruited and analyzed by next-generation sequencing to identify candidate disease-causing variants. Sanger sequencing was performed on the patients and their parents to verify the results, and short tandem repeat-based testing was conducted to confirm the biological relationship between the parents and the patients. Furthermore, cochlear implantation (CI) was performed in one AN patient to reconstruct hearing.

**Results:** Four subjects with AN were identified to share a *de novo* variant, p.E818K in the *ATP1A3* gene. Except for the AN phenotype, patients 1 and 2 exhibited varying degrees of neurological symptoms, implying that they can be diagnosed as CAPOS syndrome. During the 15 years follow-up of patient 1, we observed delayed neurological events and progressive bilateral sensorineural hearing loss in pure tone threshold (pure tone audiometry, PTA). Patient 2 underwent CI on his left ear, and the result was poor. The other two patients (patient 3 and patient 4, who were 8 and 6 years old, respectively) denied any neurological symptoms.

**Conclusion:** *ATP1A3* p.E818K has rarely been documented in the Chinese AN population. Our study confirms that p.E818K in the *ATP1A3* gene is a multiethnic cause of AN in Chinese individuals. Our study further demonstrates the significance of genetic testing for this specific mutation for identifying the special subtype of AN with somewhat favorable CI outcome and offers a more accurate genetic counseling about the specific *de novo* mutation.

## Introduction

Auditory neuropathy (AN) is a kind of hearing disorder involving different lesion sites beyond the outer hair cells (OHCs), ranging from the inner hair cells (IHCs) and synapses to auditory nerve and higher auditory centers (Rance and Starr, 2015; Moser and Starr, 2016). AN features impaired or absent response in auditory brainstem responses (ABR) and the presence of cochlear microphonics and/or detectable otoacoustic emission (OAE), showing evidence of intact outer hair cell function. Varying degrees of hearing loss are present in subjects with AN; however, their speech recognition rates are generally poor, disproportionate to the degree of hearing loss (Harrison et al., 2015; Rance and Starr, 2015; Moser and Starr, 2016). A variety of etiologies can trigger AN, generally considered to be hypoxia, infection, kernicterus, cytotoxic oncologic drugs, and genetic factors (Harrison et al., 2015).

Considering postlingual-onset AN, many syndromic forms that cause sensory and motor neuropathy have been documented, including Charcot-Marie-Tooth disease, Friedreich’s ataxia, deafness-dystonia-optic neuropathy (DDON) syndrome, autosomal dominant optic atrophy (ADOA), and AUNX1 due to *AIFM1* gene mutations in apoptosis-inducing factor (Han et al., 2017). Han et al. (2017) explored the molecular etiology of three unrelated Korean subjects manifesting AN with postlingual onset, identified a mutation in c.2452 G > A (p.E818K) of *ATP1A3* gene, and demonstrated *ATP1A3* gene to be an important and prevalent causative gene for progressive AN with postlingual onset. *ATP1A3* gene has thus been said to occur frequently *de novo* in both Caucasians and Koreans (Han et al., 2017). However, p.E818K in the *ATP1A3* gene has rarely been documented in the Chinese population.

In this study, we reported four Chinese AN patients carrying a *de novo* variant, p.E818K mutation of *ATP1A3* gene, identified through next generation sequencing (NGS). *ATP1A3* p.E818K has been generally recognized as a genetic cause of cerebellar ataxia, areflexia, pes cavus, optic atrophy, and sensorineural hearing loss (CAPOS) syndrome. A genotype–phenotype correlation study of *ATP1A3* was performed, and one AN patient underwent cochlear implantation (CI) to reconstruct hearing.

## Materials and Methods

### Ethics Statement

The study was approved by the Committee of Medical Ethics of Chinese PLA General Hospital. Written informed consent was obtained from participants.

### Subject Recruitment and Clinical Evaluations

Four AN patients at the Institute of Otolaryngology, Chinese PLA General Hospital were recruited and underwent genetic testing. The diagnostic criteria were as follows: ABR had no obvious differentiation waveform and the OAE and/or the cochlear microphonic (CM) potential could be normally extracted. Medical evidence of hearing loss, tinnitus, and other clinical abnormalities of either affected or unaffected family members was identified. Pure tone threshold (pure tone audiometry, PTA), speech discrimination score (SDS), ABR, OAE, and electrocochleogram (ECochG) were carried out to evaluate auditory status. In general, low frequencies were primarily affected; thus, we focused on the low-frequency data and calculated the PTA as the average of the thresholds of 250–1,000 Hz to avoid bias in the assessment of the degree of AN hearing loss. High-resolution computed tomography (CT) scans of the temporal bone and MRI of the internal auditory canal were performed to exclude other possible neuropathic or anatomical disorders. For patients who exhibited neurological symptoms, we carried out neurological examinations to evaluate those symptoms.

### Genetic Techniques

Next generation sequencing, including whole genome sequencing and targeted genes capture and sequencing, was performed on the four probands, Sanger sequencing was performed on the patients and their parents to verify any candidate gene variations as previously described. Variation interpretation (evaluation of the pathogenicity) was based on the standards and guidelines of the American College of Medical Genetics and Genomics and the Association for Molecular Pathology (ACMG and AMP). Short tandem repeat (STR)-based testing was conducted to confirm the biological relationship between the parents and the patients (Wang et al., 2018).

## Results

### General Clinical Information

**FIGURE 1:**
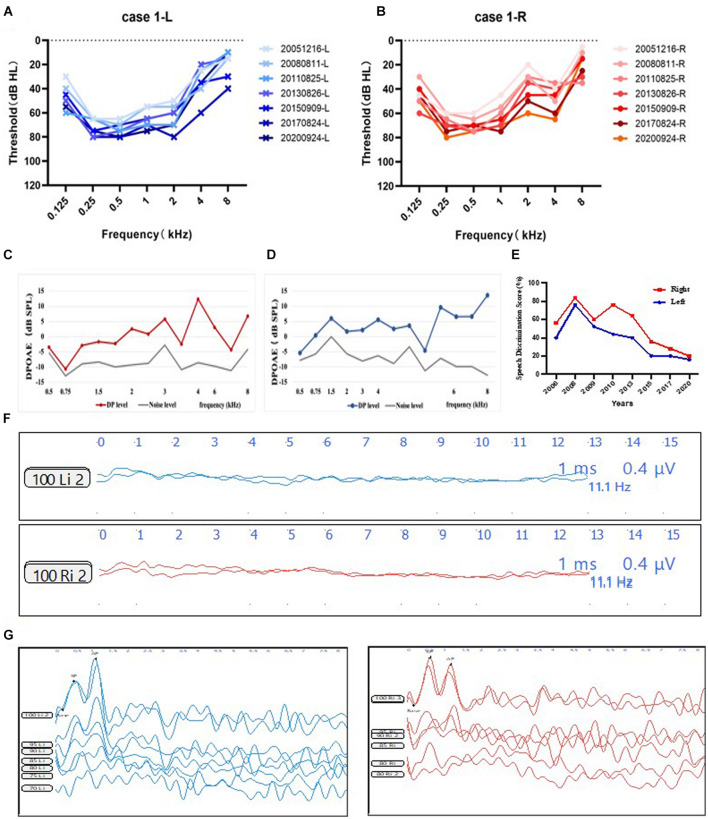
Audiological phenotype of subject 1. **(A,B)** PTA of the subject 1. Audiograms of patient 1 from 2005 to 2020 displaying moderate to severe sensorineural hearing loss, predominantly affecting the low frequencies and progressively deteriorated overtime. Blue, left ear; red, right ear. **(C,D)** Observed DPOAE responses from the right and the left ear. Red line, DPOAE level from the right ear; blue line, DPOAE level from the left ear; gray line, noise level. **(E)** SDS results from 2006 to 2020 showing decreased speech discrimination score. Blue, left ear; red, right ear. **(F)** Absent ABR waveforms at 90 dB nHL stimulus on both ears. **(G)** ECochG shows the mean ratios of -SP/AP were larger than 0.4 from both ears. Blue, left ear; red, right ear. Abbreviations: PTA, pure tone audiometry; SDS, speech discrimination score; DPOAE, distortion product optoacoustic emission; ABR, auditory brainstem response; CM, cochlear microphonic.

A total of four AN cases with *ATP1A3* variants were recruited in this study. Details on the patients in this report were collated. Clinical and audiological data were collected retrospectively. Audiological details were outlined in Figure 1 and Supplementary Figure 1.

### Detailed Medical History and Clinical Phenotype of Four Auditory Neuropathy Subjects

**FIGURE 2:**
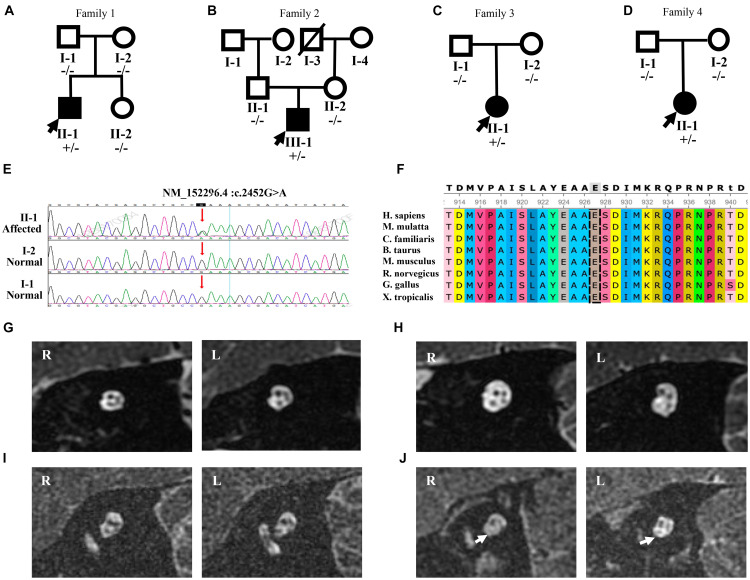
Mutation analysis and MRI results of four patients with the p.E818K mutation. **(A–C)**, and **(D)** Pedigree of four families. Filled symbols denote affected individuals, whereas unfilled symbols denote the unaffected. A slash (/) indicates a deceased person. Minus sign (-/-) represents homozygosity for the wild-type allele, and plus-minus sign (±) represents heterozygosity for the mutant allele. Probands are indicated by arrows. **(E)** Sanger sequencing chromatograms of four pedigrees confirmed that all four subjects shared one common heterozygous mutation, c.2452 G > A (arrow) leading to p.E818K amino acid change, whereas their parents are normal. **(F)** Protein conservation of no. 818 amino acid among vertebrate species (*H. sapiens*, *M. mulatta*, *C. familiaris*, *B. taurus*, *M. musculus*, *R. norvegicus*, *G. gallus*, and *X. tropicalis*). No. 818 amino acid is highly conserved. **(G–I)**, and **(J)** MRI results showing bilateral internal auditory canals (IACs) of patients 1, 2, 3, and 4, respectively. The oblique-sagittal image through the bilateral IACs of patient 4 **(J)** demonstrates absent bilateral cochlear nerves (white arrows).

Patient 1 was a 24-year-old male (Figure 2A). He exhibited dystonia and ataxia at a very early age after having a fever. At age 5, he started to suffer from visual disturbance, and at age 7, his hearing level of both ears started to deteriorate. He was diagnosed as optic atrophy at Beijing Tongren Hospital at age 8. At age 9, he was diagnosed with AN at our hospital. His sensorineural hearing loss (SNHL) matched AN, with a detectable OAE response and disappearance of the ABR response (Figures 1C,D,F), and ratios of -SP/AP were more than 0.4 in both ears (Figure 1G). His pattern visual evoked potential (P-VEP) amplitude was already reduced, indicating amblyopia found in him. Imaging tests demonstrated anatomically intact auditory nerves (Figure 2G). Somatosensory evoked potentials (SSEPs) showed prolonged latency of the bilateral tibial nerve, suggesting abnormal tibial nerve conduction. Electromyographic (EMG) signals of the upper and lower limbs did not display abnormalities. The amplitude-integrated EEG (aEEG) detected minor dysfunction. Through 15 years of follow-up from 2005 to 2020, we observed that his eyesight deteriorated over time, as did his hearing level and speech discrimination score (Figures 1A,B,E).

Patient 2 was a 22-year-old male patient (Figure 2B). He had a fever and took gentamicin at the age of 2, and he presented with bilateral hearing loss at the age of 7. His SNHL deteriorated over time, accelerating from 13 to 14 years old. Along with bilateral tinnitus, he experienced alalia as well as fatigue. However, he denied other neurologic episodes and could even ride a bicycle, although his eyesight began to decline at the age of 12. His threshold of 40 Hz AERP was 90 dB HL in both ears. Both CT scan and MRI of the internal auditory canal showed only normal findings (Figure 2H). He underwent CI on the left ear at our hospital in 2013, yet his speech perception did not improve much in 1-year follow-up period. In addition, he exhibited facial muscle twitching when the cochlear implant was used in louder environment.

Patient 3 was an 8-year-old girl (Figure 2C). She did not exhibit abnormal hearing function until the age of 7, when she suffered from a fever. Three days after her fever, she experienced hearing loss accompanied by poor speech discrimination. Her symptoms did not improve with the administration of Ginaton as well as other neurotrophic drugs, such as vitamin B12. Her PTA showed an upward sloping configuration, with an average threshold being 70 dB HL in the left ear and 61 dB HL in the right ear (Supplementary Figure 1A). In contrast, her reduction in speech recognition was drastic, being 8% in the left ear and 4% in the right ear. Her SNHL was entirely compatible with AN, considering a detectable OAE response without ABR response (Supplementary Figures 1C,E). CT scan showed no abnormality, and MRI of the internal auditory canal confirmed that she had intact cochlear nerve (Figure 2I). With no obvious neurological condition exhibited, she was diagnosed with bilateral AN.

Patient 4 was a 6-year-old girl (Figure 2D). Her syndrome of hearing loss accompanied by tinnitus appeared at age 4. Unable to benefit from the hearing aids at age 5, she came to our clinic for help. Her PTA showed an upward sloping configuration (Supplementary Figure 1B). She was diagnosed with AN with no neurological symptoms found (Supplementary Figures 1D,F). MRI result showed bilateral cochlear nerve aplasia (Figure 2J).

All the four probands passed the newborn hearing screening test successfully, suggesting that their SNHL was not congenital. Furthermore, they presented with the presence of OAEs and/or CMs, indicating preserved cochlear outer hair cell activity. However, the ABR, a sign of neural dyssynchrony, was grossly abnormal. In addition, a poor SDS was observed in all four patients, especially in noisy environments, and was disproportionate to the level of hearing loss.

### Molecular Genetic Testing and Delineation of the Causative Variant From the Four Subjects

We performed NGS, including targeted genes capture and whole genome capture, in four probands with sporadic AN and identified the most convincing causative variant that has previously been described (Kim et al., 2015). Candidate variants were validated by standard Sanger sequencing. All four AN subjects shared one heterozygous missense variant, c.2452 G > A (p.E818K) in the *ATP1A3* gene (NM_152296.4, NP_689509.1, OMIM ^∗^182350), which was located on chromosome 19q13.2 and classified as ‘‘pathogenic’’ according to CLINVAR^1^ (Landrum et al., 2016). Unaffected parents showed no variation in *ATP1A3*, indicating a *de novo* occurrence of an autosomal dominant variant, p.E818K, in all four families (Figure 2E). Evolutionary conservation analysis revealed that this variant was highly conserved throughout evolution among species (Figure 2F). STR-based testing was conducted, confirming the biological relationship between the parents and the cases and indicating that the *ATP1A3* mutation was *de novo* in nature, which strongly supports the pathogenicity. According to the ACMG guidelines, p. E818K variant met the PS2, PS3, PS4, PM2, PP1_Strong, and PP3 criteria and could be classified as pathogenic.

## Discussion

Hearing loss is the most common sensorineural disorder. It is estimated that more than half of hearing loss cases are attributable to genetic factors (He Y. et al., 2017; Wang et al., 2017; Zhu et al., 2018; Fang et al., 2019; He Z. et al., 2019; Fu et al., 2021), while the other half of hearing loss could be caused by aging, chronic infections, infectious diseases, ototoxic drugs, and noise exposure (Liu L. et al., 2016; He Z. et al., 2017; Cheng et al., 2019; Gao et al., 2019; Tan et al., 2019; Zhang et al., 2019; Zhong et al., 2020). The functions of these hearing loss genes play an essential role in the development and function of hair cells and synaptic transmission of spiral ganglion neurons (Qi et al., 2020; Qian et al., 2020; Zhang et al., 2020, 2021; Cheng et al., 2021; Lv et al., 2021). Thus, hearing loss is often induced by loss of sensory hair cells (Liu Y. et al., 2019; Qi et al., 2019; Han et al., 2020; He et al., 2020, 2021; Chen et al., 2021) and spiral ganglion neurons (Guo et al., 2016, 2019, 2020, 2021; Liu W. et al., 2019; Hu et al., 2021; Liu W. et al., 2021) in the inner ear cochlea. AN is a hearing disorder with functional outer hair cells and disrupted function of inner hair cells and/or the auditory nerve itself. Han et al. (2017) first proposed that p.E818K in *ATP1A3* gene, which encodes the NKAα3 subunit of Na, K-ATPases, can cause AN in Caucasians and Koreans, implying that *ATP1A3* serves as a global contributor to AN (Supplementary Table 1). In our study, four AN cases were tested with p.E818K in the *ATP1A3* gene. The incidence of p.E818K accounting for AN in Chinese individuals is still under investigation. Nevertheless, we found that the p.E818K mutation accounts for a high proportion of AN with postlingual onset, not only in Koreans and Caucasians but also in Chinese individuals.

*ATP1A3* p.E818K has been regarded as a causal mutation of CAPOS syndrome since identified in 2014 (Carecchio et al., 2018), long after the initial description in 1996 (Nicolaides et al., 1996). CAPOS syndrome is a rare disorder, with fewer than 100 CAPOS patients worldwide, and is sporadically reported (Demos et al., 2014; Rosewich et al., 2014; Heimer et al., 2015; Potic et al., 2015; Maas et al., 2016; Duat Rodriguez et al., 2017). Growing evidence shows that sensorineural hearing loss in CAPOS syndrome is AN, which may progress slowly over time. The typical CAPOS phenotype is characterized by acute neurological deterioration manifesting in infancy and triggered by stressful episodes, such as a febrile illness (Paquay et al., 2018). Meticulous history as well as comprehensive neurologic examination should be performed on postlingual-onset AN subjects, to detect any episode of visual disturbance and ataxia associated with CAPOS syndrome triggered by a fever that might otherwise go unnoticed. CAPOS syndrome patients initially have cochlear apical turns, manifested in a low-frequency loss in PTA. However, neuronal distribution of NKAα3 is also observed in middle and basal turns of the cochlea (Paquay et al., 2018), indicating that mid and high frequencies may also be involved. Through 15 years follow-up, we detected progressing hearing defects involving all frequencies, predominantly the low frequencies in patient 1. It is worth continuing the follow-up periods for patients with p.E818K to detect changes in PTA over time, to clarify the significance of the p.E818K mutation in AN.

In previous study, Demos et al. (2014) reported follow-up of three patients with *ATP1A3* p.E818K. All the patients showed slow progression of all symptoms with no further acute episodes. Demos et al. (2014) further reported two Caucasian families with *ATP1A3* p.E818K. In the first family, all affected suffered from acute episodic ataxic encephalopathy and/or weakness triggered by a febrile illness onset in infancy or early childhood. Recovery was not as desirable after these episodes, when variable neurologic abnormalities was lingering. All affected individuals eventually had optic atrophy and sensorineural hearing loss. One patient exhibited mild cognitive dysfunction at age 10 years. In the second family, the affected mother and her two children presented with recurrent acute episodes of neurologic disorders associated with febrile illnesses beginning in the first years of life. Features included weakness, ataxia, and a progressive decline in hearing and vision. In our study, patient 1 exhibited dystonia and ataxia at a very early age after having a fever. He exhibited optic atrophy and auditory neuropathy at an early age, which were slowly progressive over time. Follow-up period was needed for every patient to further clarify whether there was a slow progression of all symptoms, be it neurological disorders or visual and hearing impairment.

**FIGURE 3:**
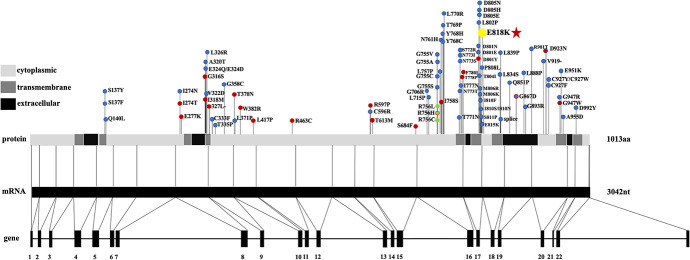
The location of AHC-causing (blue dots), RDP-causing (red dots), and CAPOS-causing (yellow dots) mutations in *ATP1A3*, mRNA, and protein. Three rare polymorphisms identified are indicated by the green dots. Mutation shared between different phenotypes is located at D923N (blue dot with a red dot inside), G867N (red dot with a blue dot inside), and R756H (red dot with a green dot inside). Different regions (cytoplasmic, transmembrane, and extracellular) of the protein are listed on the left. Abbreviations: AHC, alternating hemiplegia of childhood; CAPOS, cerebellar ataxia, areflexia, pes cavus, optic atrophy, and sensorineural hearing loss; RDP, rapid-onset dystonia Parkinsonism.

**FIGURE 4:**
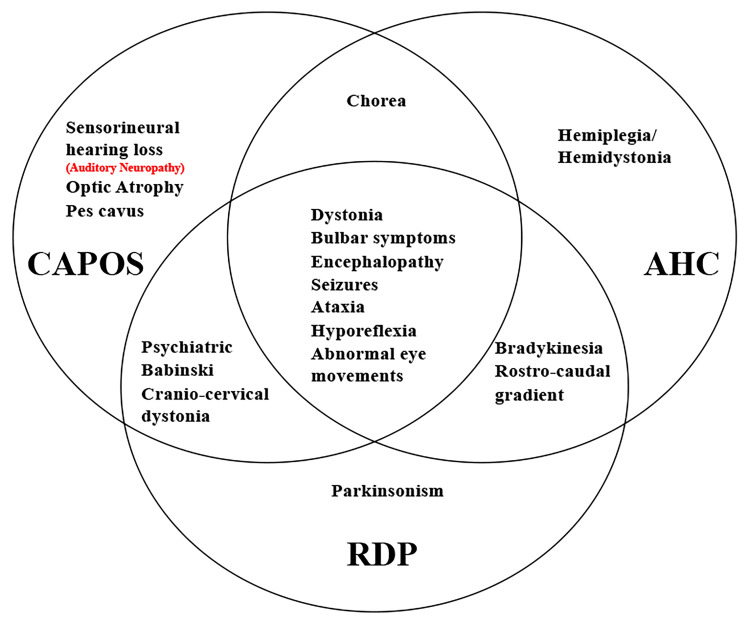
Unique and shared features between AHC, RDP, and CAPOS phenotypes. Auditory neuropathy is either a symptom of CAPOS or a new phenotype of *ATP1A3* gene.

The *ATP1A3* gene has also been reported in two other clinical entities, alternating hemiplegia of childhood (AHC, AHC2, OMIM #614820) and rapid-onset dystonia Parkinsonism (RDP, DYT12, OMIM #128235) (Blanco and Mercer, 1998; Starr et al., 2005; Rodacker et al., 2006; Blanco-Arias et al., 2009). Mutation sites are distributed across the entire coding region of *ATP1A3* in RDP and AHC (Supplementary Table 1; Clausen et al., 2017). Interestingly, all CAPOS patients reported without exception share a common mutation, p.E818K in the *ATP1A3* gene. Also, p.E818K is seemingly tightly coupled with CAPOS syndrome and has never been reported in other two disorders (Han et al., 2017; Figure 3). There is no report of hearing or visual impairment in AHC and RDP, which is vastly different from CAPOS syndrome (Figure 4; Tranebjærg et al., 2018a). However, those three entities present with an expanding phenotypic spectrum and are more often reported to share somewhat overlapping neurological phenotypes. In addition, atypical phenotypes due to *ATP1A3* gene mutations have recently been identified (Gropman et al., 2020; Guo et al., 2020). It becomes gradually clear that these overlapping phenotypes may be a series of *ATP1A3*-related symptoms, rather than allelic disorders (Carecchio et al., 2018). Moreover, the expression timing of neurological deficits of the mutation varies and has still not been fully characterized until now (Han et al., 2017). In our study, it was not very hard to observe the latency of occurrence of numerous symptoms in patients 1 and 2. Notably, patients 3 and 4 denied all neurological episodes. Continuation of the follow-up periods for these four patients is therefore necessary for the discovery of other delayed neurological symptoms. Nevertheless, whether AN is simply a symptom of CAPOS or a completely new phenotype of the *ATP1A3* gene remains to be seen.

Early diagnosis of hearing loss and timely prevention of sensory deprivation are of vital importance and confer lifelong benefits to CAPOS patients, especially for those with visual interference. The 818 position is previously confirmed located at the cytoplasmic end of transmembrane helix 6 of NKAα3, forming a salt bridge with the backbone carbonyl of Arg930, which is a residue known to stabilize the C-terminus. Tranebjærg et al. (2018b) have evaluated functional impact of Glu818Lys to the C-terminal structure and demonstrated that Glu818Lys leads to low affinity for extracellular sodium and a high rate of sodium release from the third ion-binding portion; therefore, it disrupts the function of the NKAα3 subunit of Na, K-ATPases, which is a key regulator of regaining the resting potential of the membrane after excitatory activity in the auditory pathway. In the auditory pathway, NKAα3 is expressed in the membranes of type I afferent terminals contacting the inner hair cells, spiral ganglion, and medial efferent terminals that contact the outer hair cells (Erichsen et al., 1996; McLean et al., 2009; Bottger et al., 2011), providing evidence that CI in those patients could generate likely preferable outcomes. One example of CI having an ideal outcome is CI in AN together with ADOA (OMIM #165500) owing to *OPA1* variations (Santarelli et al., 2008; Huang et al., 2009; Moser and Starr, 2016). The expression site of *OPA1* significantly overlaps with that of NKAα3, being it in the hair cells as well as in the neuronal fibers innervating the inner hair cells, ganglion cells of the cochlea, and vestibular organs (Bette et al., 2007).

Auditory neuropathy with *ATP1A3* mutation are generally considered to have ideal CI results. However, CI produces dubious outcomes in some reported cases. Tranebjaerg et al. (Fang et al., 2019) stated that four CAPOS patients received CI, two of whom gained significant benefits and the other two was relatively poor. In contrast, Atilgan et al. (2019) documented a much more beneficial effect of CI in a patient with CAPOS syndrome as compared to patients with SNHL. These findings could be explained by the fact that the *ATP1A3* gene is expressed not only in synapse but also in postsynaptic spiral ganglion cells (SGNs). If the lesion is mainly at the synapse, good CI results can be expected, similar to those in *OPA1* variants (Han et al., 2017), whereas CI is less useful with lesions dominantly at the SGNs. In fact, in most postsynaptic AN, CI has a dubious outcome (Supplementary Table 2). An example is CI in the SGNs (Fasquelle et al., 2011; Eppsteiner et al., 2012; Atilgan et al., 2019). CI performed earlier in *TMPRSS3* generated a preferable outcome, while the outcomes in later implantees were not as favorable (Chung et al., 2014). Patients who benefit most from CI were reported to be relatively younger (Han et al., 2017; Tranebjærg et al., 2018b), yet an accurate CI efficacy estimation with age and other possible impact factors taken into consideration is still awaiting discussion. Chaudhry et al. (2020) summarized 25 postsynaptic CI results, with 22 displaying modest to significant benefit, and demonstrated that CI behaves variably but generally good in postsynaptic AN. Moreover, the best functional outcome of CI observed at the short-term follow-up may decrease over time. Although earlier electrical stimulation of SGNs may attenuate the degeneration of the neurons, progressive degeneration of SGNs could possibly occur due to a disrupted function of NKAα3 (Han et al., 2017). In our study, patient 2 underwent CI on his left ear, but the result was hardly desirable. One possible explanation is that his age by the time he received surgery and the duration of his hearing loss are too long to ensure SGN activities. Moreover, the result of CI may be in relation with the characteristic of the PTA of the patients. Therefore, a continuous follow-up period for CI performance in those patients is warranted, to better determine if the current benefits from CI decreases over time.

The identification of p.E818K in *ATP1A3* has implications for future risk calculation and genetic counseling. In CAPOS families with an autosomal dominant inheritance pattern, the chance of offspring carrying pathogenic mutations is 50%, with varying degrees of penetrance. In addition, all reported CAPOS patients had unaffected siblings without exception, revealing that all pedigrees of CAPOS syndrome are sporadic cases. It is still unclear why *de novo* variants frequently occur in *ATP1A3.* The age of the father has been reported to be a strong impactor of *de novo* mutations (Niederberger, 2013). However, that study failed to explain all *de novo* cases. A genetic context in *ATP1A3* that favors the occurrence of *de novo* variants may account for this phenomenon, awaiting further clarification.

Here, four AN patients were identified with p.E818K mutation in the gene *ATP1A3*, causing progressive AN with postlingual onset and varying degrees of syndromic features. Our study confirms that *ATP1A3* in Chinese individuals is undoubtedly an important genetic cause of progressive AN with postlingual onset. Genetic testing for this specific mutation site allows for identification of a special subtype of AN with somewhat preferable CI results, as well as accurate genetic counseling.

## Data Availability Statement

The data presented in the study are deposited in the ClinVar repository, accession number VCV000156238.

## Ethics Statement

The studies involving human participants were reviewed and approved by Committee of Medical Ethics of Chinese PLA General Hospital. Written informed consent to participate in this study was provided by the participants’ legal guardian/next of kin. Written informed consent was obtained from the individual(s), and minor(s)’ legal guardian/next of kin, for the publication of any potentially identifiable images or data included in this article.

## Author Contributions

HW and WW conceived and designed the experiments and wrote the manuscript. HW, WW, JL, LX, LL, and FX performed the experiments. HW analyzed the data. JL, LL, LX, and FX contributed to the reagents, materials, and analysis tools. HW, JG, and QW critically read and discussed the manuscript. All authors contributed to the article and approved the submitted version.

## Conflict of Interest

The authors declare that the research was conducted in the absence of any commercial or financial relationships that could be construed as a potential conflict of interest.

## Publisher’s Note

All claims expressed in this article are solely those of the authors and do not necessarily represent those of their affiliated organizations, or those of the publisher, the editors and the reviewers. Any product that may be evaluated in this article, or claim that may be made by its manufacturer, is not guaranteed or endorsed by the publisher.

## Footnotes

1
https://www.ncbi.nlm.nih.gov/clinvar/

## Supplementary Material

Supplementary Figure 1 Audiological phenotype of subject 3 and 4. **(A,B)** PTA of subjects 3 and 4. Blue, left ear; red, right ear. **(C,D)** Observed DPOAE responses from the right and the left ear. Red line, DPOAE level from the right ear; blue line, DPOAE level from the left ear; gray line, noise level from both ears. **(E,F)** Absent ABR waveforms at 90 dB nHL stimulus on both ears of subjects 3 and 4. Blue, left ear; red, right ear.

Supplementary Table 1 *ATP1A3* genotypes as they correlate with RDP, AHC, and CAPOS phenotypes.

Supplementary Table 2 Postsynaptic auditory neuropathies and their CI outcomes.
