# Supplementary material for: Auditory Neuropathy as the Initial Phenotype for Patients With ATP1A3 c.2452 G > A: Genotype–Phenotype Study and CI Management
Source: Front Cell Dev Biol. 2021 Oct 8;9:749484. doi: 10.3389/fcell.2021.749484 (PMC8531511; doi:10.3389/fcell.2021.749484)
Supplement: Supplementary Figure 1 — Audiological phenotype of subject 3 and 4. (A,B) PTA of subjects 3 and 4. Blue, left ear; red, right ear. (C,D) Observed DPOAE responses from the right and the left ear. Red line, DPOAE level from the right ear; blue line, DPOAE level from the left ear; gray line, noise level from both ears. (E,F) Absent ABR waveforms at 90 dB nHL stimulus on both ears of subjects 3 and 4. Blue, left ear; red, right ear. [file Data_Sheet_1.docx]

Supplementary Material


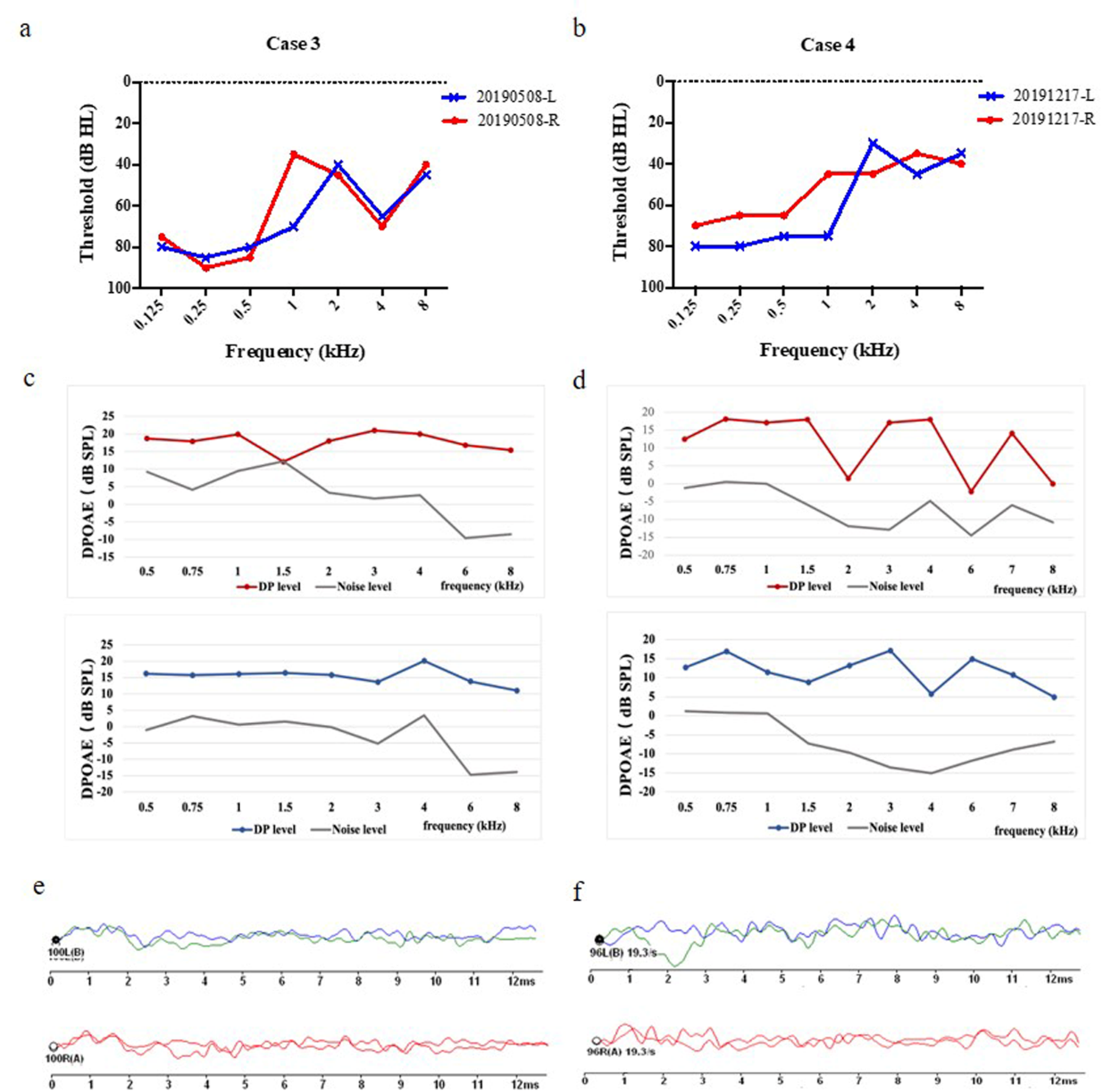


**Supplementary Figure 1.** Audiological phenotype of subject 3 and 4. (a, b) PTA of subject 3 and 4 showing moderate to severe sensorineural hearing loss, predominantly affecting the low frequencies. Blue, left ear; Red, right ear. (c) Observed DPOAE responses from both ears of subject 3. (d) Observed DPOAE responses from both ears of subject 4. (e) Absent ABR waveforms at 100 dB nHL stimulus on both ears of subject 3. (f) Absent ABR waveforms at 100 dB stimulus on both ears of subject 4.

|  | | | Supplement Table 1. ATP1A3 genotypes as they correlate with RDP, AHC, and CAPOS phenotypes. | | | | | | |
| --- | --- | --- | --- | --- | --- | --- | --- | --- | --- |
| Nucleotide Change | Exon | | | Protein Change | Phenotype | Inheritance | Recurrence (Yes/No), Frequency | Origin | References/Last reviewed |
| 385G>A | | 5 | | V129M | Juvenile onset psychosis | N/A | N/A | American | Apr 11, 2016 |
| 410C>A | | 5 | | S137Y | AHC | Sporadic | Yes, infrequent | American | Oct 21, 2019 |
| 410C>T | | 5 | | S137F | AHC | Sporadic | No | American | Aug 19, 2019 |
| 419A>T | | 5 | | Q140L | AHC | Sporadic | No | American | May 4, 2014 |
| 821T>A | | 8 | | I274N | AHC | Sporadic and familial(dominant) | Yes, infrequent | Germany | May 4, 2014 |
| 821T>C | | 8 | | I274T | RDP | Sporadic | No | American | Linazasoro et al.,2002 |
| 829G>A | | 8 | | E277K | RDP | Sporadic | Yes, infrequent | American | Zaremba et al.,2004 |
| 946G>A | | 8 | | G316S | RDP | N/A | N/A | American | Apr 7, 2017 |
| 954C>G | | 8 | | I318M | RDP | N/A | N/A | N/A | Jun 2, 2020 |
| 958G>A | | 8 | | A320T | AHC | Sporadic | No | English | Trump et al.,2016 |
| 965T>A | | 8 | | V322D | AHC | Sporadic | Yes, infrequent | Germany | May 4, 2014 |
| 967C>T | | 8 | | P323S | not provided | N/A | N/A | N/A | May 5, 2015 |
| N/A | | 8 | | E324Q | AHC | N/A | N/A | France | Eleni Panagiotakaki et al. 2015 |
| 972G>C | | 8 | | E324D | AHC | N/A | N/A | France | [Viollet, 2015](http://www.ncbi.nlm.nih.gov/sites/entrez?cmd=Retrieve&db=PubMed&list_uids=25996915&dopt=Abstract) |
| 977T>G | | 8 | | L326R | AHC | N/A | N/A | Japanese | [Viollet, 2015](http://www.ncbi.nlm.nih.gov/sites/entrez?cmd=Retrieve&db=PubMed&list_uids=25996915&dopt=Abstract) |
| 976_978delCTG | | 8 | | L327del | RDP | Sporadic | No | N/A | May 4, 2014 |
| 998G>T | | 9 | | C333F | AHC | Sporadic | Yes, infrequent | American | May 4, 2014 |
| 1003A>C | | 9 | | T335P | AHC | Sporadic | No | Germany | May 4, 2014 |
| 1072G>T | | 9 | | G358C | AHC | Sporadic | No | Japanese | May 4, 2014 |
| 1109C>A | | 9 | | T370N | RDP | N/A | N/A | Germany | May 4, 2014 |
| 1112T>C | | 9 | | L371P | AHC | Sporadic | No | Germany | May 4, 2014 |
| 1144T>C | | 9 | | W382R | RDP | N/A | N/A | Germany | May 4, 2014 |
| 1250T>C | | 10 | | L417P | RDP | N/A | N/A | Germany | May 4, 2014 |
| 1387C>T | | 11 | | R463C | RDP | N/A | N/A | N/A | Dec 31, 2019 |
| 1786T>C | | 13 | | C596R | AHC | N/A | N/A | Japanese | [Viollet, 2015](http://www.ncbi.nlm.nih.gov/sites/entrez?cmd=Retrieve&db=PubMed&list_uids=25996915&dopt=Abstract) |
| 1790G > C | | 13 | | R597P | RDP | Sporadic | N/A | Germany | G.R. Wenzel, et al. 2017 |
| 1838C>T | | 14 | | **T613M*** | RDP | Sporadic and familial(dominant) | Yes, most frequent recurrence in RDP, n=6 | American | Dobyns et al., 1993;  Brashear et al.,1997 |
| 2051C>T | | 15 | | S684F | RDP | N/A | N/A | Serbia | May 4, 2014 |
| 2116G>A | | 15 | | G706R | AHC | N/A | N/A | Chinese | Oct 10, 2017 |
| N/A | | 15 | | L715P | AHC | Sporadic | N/A | France | Eleni Panagiotakaki et al. 2015 |
| 2263G>A | | 16 | | G755S | AHC | Sporadic | Yes, infrequent | American | May 4, 2014 |
| 2263G>T | | 16 | | G755C | AHC | Sporadic | Yes, infrequent | Germany | Sep 6, 2019 |
| 2264G>C | | 16 | | G755A | AHC | N/A | N/A | Japanese | May 4, 2014 |
| 2263G>T | | 16 | | G755V | AHC | Sporadic | N/A | Germany | Hendrik Rosewich, 2012 |
| 2266C>T | | 16 | | R756L | FIPWE | Sporadic and familial | Yes, infrequent | Japanese | Hully M,2017 |
| 2267G>A | | 16 | | R756H | RDP | Sporadic | No | American | Brashear,2012; Hully M,2017 |
| 2266G>T | | 16 | | R756C | FIPWE | Sporadic and familial | Yes, infrequent | Japanese | Hully M,2017 |
| 2270T>C | | 16 | | L757P | AHC | Sporadic | No | Germany | May 4, 2014 |
| 2273T>G | | 16 | | I758S | RDP | Familial(dominant) | No | American | May 4, 2014 |
| 2281A>C | | 17 | | N761H | AHC | Sporadic | No | Chinese | May 21, 2015 |
| 2302T>C | | 17 | | Y768H | AHC | Sporadic | No | Chinese | May 21, 2015 |
| 2303A>G | | 17 | | Y768C | AHC | Sporadic | No | Chinese | May 21, 2015 |
| 2305A>C | | 17 | | T769P | AHC | Sporadic | No | Chinese | May 21, 2015 |
| N/A | | 17 | | L770R | AHC | Sporadic | No | Chinese | May 19, 2014 |
| 2312C>A | | 17 | | T771N | AHC | Sporadic | No | Chinese | May 4, 2014 |
| 2312C>T | | 17 | | T771I | Capos | N/A | N/A | Japanese | Mar 2, 2020 |
| 2316C>A | | 17 | | S772R | AHC | Sporadic | No | Germany | May 4, 2014 |
| 2316C> G | | 17 | | S772R | AHC | Sporadic | No | Chinese | May 19, 2014 |
| 2318A>G | | 17 | | N773S | AHC | Sporadic | No | Germany | Jan 1, 2019 |
| 2318A>T | | 17 | | N773I | AHC | Sporadic | No | American | May 4, 2014 |
| 2318A>C | | 17 | | N773T | AHC | Sporadic | No | Chinese | May 19, 2014 |
| 2330T>A | | 17 | | I777N | AHC | N/A | N/A | N/A | Oct 12, 2018 |
| 2332A>C | | 17 | | T778P | RDP | N/A | N/A | N/A | Jan 16, 2020 |
| 2338T>C | | 17 | | F780L | RDP | Familial(dominant) | No | American | May 4, 2014 |
| 2401G>A | | 17 | | D801N | AHC/RDP  /Capos | Sporadic | Yes, frequent in AHC, n>60 reported | Germany | Mar 09, 2015 |
| 2401G>T | | 17 | | D801Y | RDP | Familial | No | American | May 4, 2014 |
| 2403T>A | | 17 | | D801E | AHC | Sporadic | N/A | Denmark | Apr 16, 2018 |
| 2401G>A | | 17 | | D801N | AHC | Sporadic | N/A | France | Eleni Panagiotakaki et al. 2015 |
| 2405T>C | | 17 | | L802P | AHC | Sporadic | N/A | Chinese | May 19, 2014 |
| 2411C>T | | 17 | | T804I | AHC | Sporadic | Yes, infrequent | Spain | May 4, 2014 |
| 2413G>A | | 17 | | D805N | AHC | Sporadic | No | Chinese | May 21, 2015 |
| 2413 G >C | | 17 | | D805H | AHC | Sporadic | N/A | Chinese | May 19, 2014 |
| 2415C>G | | 17 | | D805E | AHC | Sporadic | N/A | Germany | Mar 09, 2015 |
| 2417T>G | | 17 | | M806R | AHC | Sporadic | No | American | Nov 22,2017 |
| 2417T>A | | 17 | | M806K | AHC | Sporadic | N/A | Chinese | May 19, 2014 |
| 2423C>T | | 17 | | P808L | AHC | Sporadic | N/A | Chinese | May 19, 2014 |
| 2428A>T | | 17 | | I810F | AHC | Sporadic | No | Germany | May 4,2014 |
| 2429T>G | | 17 | | I810S | AHC | Sporadic | No | American | May 4,2014 |
| 2429T>A | | 17 | | I810N | AHC | Sporadic | N/A | Chinese | May 19, 2014 |
| 2431T>C | | 17 | | S811P | AHC | Sporadic | Yes, infrequent | American | May 14,2019 |
| 2443G>A | | 18 | | E815K | AHC/  RDP/  Capos | Sporadic | Yes, frequent in AHC, n>40 reported | Germany | Jan 01, 2017 |
| 2452G>A | | 18 | | E818K | Capos | Familial(dominant) | Yes, 100% so far | Canada | Kyu-Hee Han，2017 |
| 2501T>C | | 18 | | L834S | AHC | Sporadic | No | Chinese | May 21, 2015 |
| 2516T>C | | 18 | | L839P | AHC | Sporadic | No | Chinese | May 21, 2015 |
| 2542+1G>A | | Intron 18 | | Splice site | AHC | Sporadic | Yes, infrequent | N/A | May 4,2014 |
| 2552 A>C | | 19 | | Q851P | AHC | Sporadic | No | Chinese | May 21, 2015 |
| 2600G>A | | 19 | | G867D | RDP/AHC | Sporadic | No | Germany | Brashear A, 2018 |
|  | |  | | L888P | AHC | Sporadic | N/A | France | Eleni Panagiotakaki et al. 2015 |
| .2677G>A | | 19 | | G893R | AHC | Sporadic | N/A | Chinese | May 19, 2014 |
| 2702G>C | | 20 | | R901T | AHC | Sporadic | N/A | Germany | Mar 09, 2015 |
| 2755_2757delGTC | | 20 | | V919del | AHC | Sporadic | No | N/A | Heinzen EL, 2012 |
| 2767G>A | | 20 | | D923N | AHC/RDP | Familial (AHC), sporadic and familial (RDP) | Yes, low frequency, n=5 | Germany | Jun 13, 2019 |
| 2780G>A | | 20 | | C927Y | AHC | Sporadic | No | Japanese | Brashear A, 2018 |
| 2780G>T | | 20 | | C927F | AHC | Sporadic | Yes, infrequent | Japanese | May 4, 2014 |
| 2781 C> G | | 20 | | C927W | AHC | Sporadic | No | Spain | Oct 31,2013 |
| 2780 G>T | | 20 | | C927F | AHC | Sporadic | No | Japanese | Masayuki Sasaki, 2014 |
| 2780 G>A | | 20 | | C927Y | AHC | Sporadic | No | Japanese | [Atsushi Ishii](https://www.ncbi.nlm.nih.gov/pubmed/?term=Ishii%20A%5BAuthor%5D&cauthor=true&cauthor_uid=23409136),2013 |
| 2839G>A | | 20 | | G947R | AHC | Sporadic | Yes, third most frequent for AHC in European Caucasian cohorts, n>10 | American | May 30,2019 |
| 2839G>T | | 20 | | G947W | RDP/  AHC | Sporadic | N/A | American | May 30, 2019 |
| 2839G>C | | 20 | | G947R | AHC | N/A | Yes, low frequency, n=4 | American | May 4,2014 |
| 2851G>A | | 20 | | E951K | AHC | Sporadic | N/A | Germany | Mar 09, 2015 |
| 2864C>A | | 20 | | A955D | AHC | Sporadic | No | American | May 4,2014 |
| 2974G>T | | 22 | | D992Y | AHC | Sporadic | Yes, infrequent | American | May 4,2014 |

N/A, not applicable.

Supplementary Table 2. Postsynaptic auditory neuropathies and their CI outcomes. CI in *ATP1A3* mutants generally produce good results.

| **Gene** | **Name of condition/syndrome** | **Phenotype** | **CI outcome** | **References** |
| --- | --- | --- | --- | --- |
| *ATP1A3* | Cerebellar ataxia-areflexia-pes cavus-optic atrophy-sensorineural hearing loss (CAPOS syndrome) | Slowly progressive sensorineural hearing loss; Optic atrophy; Acute episodes of neurological deterioration; Ataxia; Areflexia | Good/Poor | Demos MK et al, 2014; **This study** |
| *AIFM1* | X-linked auditory neuropathy; Cowchock Syndrome | Familial and sporadic ANSD. Mental retardation, Motor dysfunction and muscle weakness | Poor | Zong L et al, 2015 |
| *DIAPH3* | Autosomal dominant NSHL | Moderate to profound deafness | Good | Starr A et al, 2004; Schoen CJ et al, 2010 |
| *FXN* | Friedreich’s ataxia (FRDA) | Ataxia; Optic neuropathy; Axonal neuropathy; normal hearing threshold; Hypertrophic cardiomyopathy; Mild deafness | Modest | Kwei KT et al, 2020 |
| *NARS2* | Autosomal recessive NSHL; Leigh syndrome | Absent ABRs, present CM, and absent OAEs | N/A | Simon M et al, 2015 |
| *OPA1* | Syndromic dominant optic atrophy (DOA+) | Optic atrophy as well as auditory neuropathy presenting with moderate to severe hearing loss | Good | Lenaers G et al, 2012 |
| *PM22* (CMT 1A); *MPZ* (CMT 1B) | Charcot-Marie-Tooth disease (CMT) | Mild to severe deafness; Demyelinating neuropathy | Good | Stojkovic T et al, 2016; Kovach MJ et al, 2002 |
| *ROR1* | Common cavity malformation and auditory neuropathy | Autosomal recessive deafness associated with a common cavity inner ear malformation | N/A | Diaz-Horta O et al, 2016 |
| *SLC52A3*; *SCL52A2*; *SCL52A1* | Brown-Vialetto-Van-Laere syndrome (BVVL) | Progressive pontobulbar palsy; Sensorineural deafness; Facial weakness; Respiratory compromise | Good | Bosch AM et al, 2012; Sathasivam S et al, 2008 |
| *TIMM8A* | Deafness-dystonia-optic neuronopathy (DDON) syndrome | Childhood onset auditory neuropathy; Slowly progressive dystonia and ataxia; Decreased visual acuity; Dementia | Poor | Tranebjærg L et al, 2003 |

N/A, not applicable.
